# Topical application of the plant extract SDTL-E in ovariectomized rats: A potential new approach for treating osteoporosis

**DOI:** 10.3389/fmed.2022.988235

**Published:** 2022-10-20

**Authors:** Hui-Yuan Shih, Jun-Hua Lu, Ai-Hua Xiong, Juliana Man-Wai Tse, Ben Siu-Tak Wong

**Affiliations:** ^1^Hong Kong Small Biomolecules Laboratory, Hong Kong, Hong Kong SAR, China; ^2^Department of Pharmacology, Jinan University College of Pharmacy, Guangzhou, China; ^3^Laboratory Technical Teaching Centre, Jinan University College of Pharmacy, Guangzhou, Hong Kong SAR, China; ^4^SDTL Reborntech Company Limited, Hong Kong, Hong Kong SAR, China

**Keywords:** ovariectomy (OVX), calcium, trabecular bone, bone strength, estradiol, plant extract, topical application, osteoporosis

## Abstract

Current osteoporosis medications have drawbacks of causing side effects and having slow onset, therefore developing osteoporosis drugs with faster onset and less side effects is essential. This study investigated the effects of the natural plant extract, SDTL-E, in ovariectomized (OVX)-induced osteoporosis rats. Rats were randomly assigned to sham operation control group (Control Group); OVX rat model group (Model Group) or OVX rat SDTL-E treatment group (SDTL-E Group). All groups underwent ovariectomy, but the Control Group did not have the ovaries removed. SDTL-E Group was treated with SDTL-E, Model and Control Groups were treated with vegetable oil, treatments were topically applied twice daily for 20 days. Results showed when compared with Model Group, SDTL-E Group significantly restored serum estradiol back to near Control Group level, serum ALP activity, serum and urinary calcium were significantly decreased, bone mechanics indicators increased and trabecular bone numbers slightly increased. These results demonstrated 20 days of SDTL-E topical treatment improved bone strength and trabecular bone structure in OVX-induced osteoporosis rats. The underlying mechanisms include restoring estradiol level, reducing bone turnover, net bone resorption, bone calcium loss, and calcium excretion through kidney. These findings suggest topical application of plant extract is a potential new approach with quick efficacy for treating osteoporosis.

## Introduction

Osteoporosis is a worldwide bone disease having a prevalence of 18.3% (1), affecting around 1.36 billion people out of 7.9 billion global population. Among them, elderly women are at a higher risk. Prevalence of osteoporosis in women over 50 years of age is 37% (2), four times higher than that of men (3). When women reach menopause, estrogen deficiency impairs the normal bone turnover cycle. Decrease in estrogen leads to bone resorption process exceeding bone formation process, resulting in a net loss of bone (4), which increases fracture risk. Osteoporotic fractures are associated with increased mortality rate by 15–20% within 1 year, and a 2.5-fold increased future fracture risk (5). The main objective of treating osteoporosis is to reduce bone loss for lowering fracture risk and to reduce post-fracture mortality risk (6), therefore, it is important for osteoporosis drugs to efficiently lower the fracture rate and post-fracture mortality rate.

Current osteoporosis treatments are mainly administered orally or through injection and there are two types of treatment, the first type is antiresorptive treatment, which decreases the rate of bone resorption, drugs belonging to this type include bisphosphonates, estrogen, and denosumab. The second type is anabolic treatment, which increases bone formation, such as parathyroid hormone analogs (7). The treatment duration of the above osteoporosis drugs can range from 12 to 36 months (8–11), but cannot be taken for a long term due to their various side effects (12–15), seriously decreasing the efficacy of treatment and hindering the reduction of post-fracture mortality risk. Furthermore, a study pointed out that 30% of post-hip fracture deaths occurred within 6 months after fracture (16), indicating that current osteoporosis drugs have serious drawbacks of having a treatment duration much longer than the time for post-fracture deaths to occur. Therefore, it is urgent and essential to develop new osteoporosis drugs with faster onset, shorter treatment duration, better efficacy and less side effects to reduce fracture risk and post-fracture mortality risk.

SDTL-E is a patented natural plant extract (US provisional patent application number: 63/195054). A review pointed out that many natural plants have long been used as herbal medicines to prevent and treat osteoporosis. Main common active ingredients in the plants, such as flavonoids and luteolin, increase osteoblast activity through estrogenic effects, and may also promote osteoblast activity and suppress osteoclast activity through modulating cytokines, and regulating biochemical pathways, such as Wnt/β-catenin, and RANKL/RANK/OPG pathway to achieve their anti-osteoporotic effects (17). Herbal medicines are generally considered natural and safe, however, they may cause hepatotoxicity (17), which is a result of first pass metabolism of the liver when herbal medicine is taken orally (18). To avoid first pass metabolism and hepatotoxicity, topical application route can be considered as it was documented in Ayurveda that the paste of stem of medicinal plants can be applied topically for treatment of osteoporosis (19).

In this study, SDTL-E was topically applied for 20 days to the commonly used ovariectomized (OVX)-induced osteoporosis rat model (20). Then changes of estradiol, various bone turnover markers such as serum alkaline phosphatase (ALP) activity (21), and serum and urinary calcium (22), bone mineral density (BMD), various bone mechanics indicators and bone histology were assessed, so as to understand the mechanism and therapeutic efficacy of SDTL-E on the treatment of osteoporosis.

## Materials and methods

### Animals and experimental groups

Three-month-old Sprague Dawley female rats were purchased from Guangdong Medical Laboratory Animal Centre (Foshan, Guangdong, China). They were maintained with free access to bottled tap water and were fed with specific-pathogen-free grade whole granular rat feed (Guangdong Medical Laboratory Animal Centre) in a 12-h light–dark cycle under an environment with temperature of 20^°^C and relative humidity of 80%. Rats were maintained for 15 days before performing further procedures. All procedures performed were approved by the Laboratory Animal Ethics Committee of Jinan University and were in accordance with Animal Care Ethical Guidelines of Jinan University. Rats were randomly assigned to one of the following three treatment groups (10–12 rats per group): (1) sham operation control group (Control Group); (2) OVX rat model group (Model Group); (3) OVX rat model SDTL-E treatment group (SDTL-E Group).

### Ovariectomized rat model and treatment

Model Group and SDTL-E Group rats were anesthetized with ethyl ether (Sinopharm Chemical Reagents, Shanghai, China) under sterile environment, then underwent surgery where an incision of approximately 1.5 cm was made on the abdomen 4 cm below the xiphoid. The fat pads were then pulled aside, and the ovaries were removed, followed by repositioning of the fat pads and suturing the incision. The Control Group rats underwent the above surgery but without removing the ovaries. Intramuscular injection of ampicillin (Sinopharm Chemical Reagents) (100,000 units/rat) were then given to the rats for 3 days. The rats were maintained for 3 months, then were weighted and examined by dual-x-ray densitometry to confirm the successful establishment of OVX-induced osteoporosis rat model.

The rats were then anesthetized by injecting 3% pentobarbital sodium (36 mg/kg) (Sinopharm Chemical Reagents), followed by removing an area of back hair of about 3 cm × 4 cm by smearing with hair removing agent (sodium sulfide 3 g, soap powder 1 g, starch 7 g, add water until paste is formed) on the back for 5 min. After 48 h, 100 μL/kg/day SDTL-E or vegetable oil (Shandong Luhua Group, Yantai, Shandong, China) was topically applied to the hair removed area of the rats twice a day for 20 days. Control Group and Model Group were treated with vegetable oil, while SDTL-E Group was treated with SDTL-E.

Urine was collected for 24 h on day 19 and stored at −20^°^C for subsequent examination. 45 min after the last treatment, rats were anesthetized by 3% pentobarbital sodium. Blood was extracted from the abdominal aorta and centrifuged at 3,000 rpm, then the supernatant was collected and stored at −80^°^C for further examination. Rats were then sacrificed by neck dislocation, followed by surgically removing the right femur, tibia and left femur, which were stored at −80^°^C wrapped with gauze soaked in physiological saline (Sinopharm Chemical Reagents) for further examination.

### Serum alkaline phosphatase activity assay

Serum ALP activity was measured by ALP Assay Kit (Nanjing Jiancheung Bioengineering Institute, Nanjing, Jiangsu, China) according to the manufacturer’s instructions. Serum ALP in the samples reacted with the substrate, p-nitrophenolphosphate, provided by the kit to generate a yellow product, which was quantified through measuring absorbance at a wavelength of 415 nm by S22PC Spectrophotometer (Shanghai Lengguang Technology Co., Ltd., Shanghai, China) to determine serum ALP activity.

### Calcium assay

Serum and urinary calcium ion levels were measured by Calcium Assay Kit (Nanjing Jiancheung Bioengineering Institute) according to the manufacturer’s instructions. Calcium ions in the samples reacted with the substrate, Methylthymol blue, provided by the kit to generate a blue product, which was quantified through measuring absorbance at a wavelength of 610 nm by S22PC Spectrophotometer to determine serum and urinary calcium ion levels.

### Creatinine assay

Urinary creatinine level was measured by Creatinine Assay Kit (Nanjing Jiancheung Bioengineering Institute) according to the manufacturer’s instructions. Urinary creatinine in the samples was oxidized by the oxidase provided by the kit resulting in a purplish red product, which was quantified through measuring absorbance at a wavelength of 546 nm by S22PC Spectrophotometer to determine urinary creatinine level.

### Estradiol assay

Serum estradiol level was measured by AxSYM Estradiol Assay (Abbott, Chicago, Illinois, U.S.A) according to the manufacturer’s instructions. Serum estradiol in samples were bound to matrix cell as antibody-estradiol-alkaline phosphatase conjugate, followed by reaction with the substrate 4-methylumbelliferyl phosphate to generate fluorescence, which was detected and quantified by ARCHITECT i2000SR immunoassay analyzer (Abbott) to determine serum estradiol level.

### Bone densitometry

Rats were anesthetized with ethyl ether. When the rats were in a stable lethargic state for more than 5 min, they were placed under the Lunar Prodigy Dual-X ray Densitometer (GE Healthcare, Chicago, Illinois, U.S.A), and whole-body scan was performed at 60.0 mm/s, with 1.0 × 1.0 mm step size using the calibrated small-animal software to determine the BMD (CV < 1%).

### Bone mechanics test

The distal and proximal ends of the right tibia were embedded in polymethyl methacrylate and then mounted onto a combined-axial-motion and torsional-testing jig, which was attached to the 855 Mini Bionix testing system (MTS Systems, Eden Prairie, Minnesota, U.S.A) for bone torsional testing. The distal end of the specimen was rotated laterally at 6°/min until bone failure was observed. The load displacement curves were recorded and the following parameters were calculated: torsion power (N), torque (Nmm), shear stress (MPa), and shear modulus (MPa).

### Histological examination

All the soft tissues of the proximal half of the left femur were removed, freshly prepared 10% neutral buffered formalin (Sinopharm Chemical Reagents) was used to fix the bone for 24 h. The bone was then rinsed with 0.01 M phosphate buffered saline (Sinopharm Chemical Reagents). After that, the bone was decalcified by 20% formic acid (Sinopharm Chemical Reagents) for 6 days, with the formic acid changed daily. Then the bone was rinsed with 0.01 M phosphate buffered saline, and dehydrated with 75, 80, 95, and 100% ethanol (Sinopharm Chemical Reagents) successively, followed by treating 15 min in xylene (Sinopharm Chemical Reagents) twice. After that, the bone was soaked in a 60^°^C paraffin bath for 3 h and embedded overnight. On the next morning, 5 μm thick cross sections were prepared on a polylysine-treated glass slide. The glass slide was dried, followed by hematoxylin and eosin staining (Sinopharm Chemical Reagents) and changes in bone structure were examined under a light microscope (Leica, Wetzlar, Germany).

### Statistical analysis

All data were analyzed by the statistical software GraphPad Prism 9 (GraphPad Software Inc., La Jolla, CA, U.S.A.). Results are expressed as mean ± standard deviation (*SD*). Multiple comparisons between groups were analyzed by one-way ANOVA with Newman–Keuls *post-hoc* test and were considered statistically significant when *p* < 0.05.

## Results

### Reduced bone mineral density in ovariectomized-induced osteoporosis rat model

Three months after Model Group and SDTL-E Group rats were OVX, BMD was measured before treatment. The BMD of Model Group and SDTL-E Group were significantly lower than the Control Group (*p* < 0.05) (Table 1), indicating the OVX-induced osteoporosis rat model was successfully established.

**TABLE 1:** BMD of rats 3 months after ovariectomy before treatment by bone densitometry analysis.

Groups	(*n*)	BMD (g/cm^2^)
Model	12	0.20 ± 0.02*
Control	12	0.22 ± 0.02
SDTL-E	10	0.20 ± 0.01*

Values are mean ± *SD*.

*p < 0.05 vs. Control Group.

### Body weight changes of ovariectomized-rats

**FIGURE 1:**
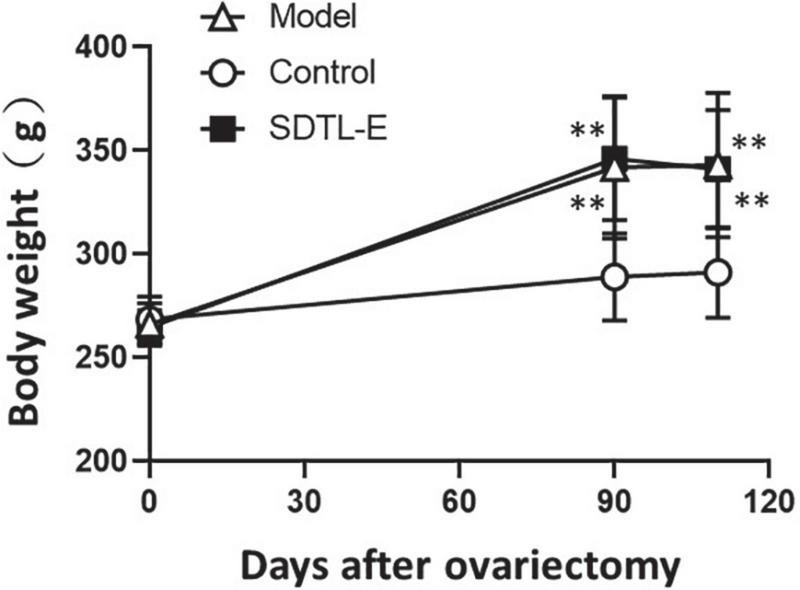
Changes of body weight of rats. 90 days after ovariectomy, Model Group (*n* = 12) and Control Group (*n* = 12) rats were treated with vegetable oil. SDTL-E Group (*n* = 10) rats were treated with SDTL-E. All treatments were topically applied twice daily for 20 days. Rats were weighed before ovariectomy, 90 days after ovariectomy (treatment day 1) and 110 days after ovariectomy (treatment day 20). Values are mean ± *SD*. ^**^p < 0.01 vs. Control Group.

Rats of all groups had similar body weight before ovariectomy. The body weight of Model Group and SDTL-E Group were significantly higher than the Control Group (*p* < 0.01) after treatment day 1, which was 3 months after ovariectomy. SDTL-E treatment on SDTL-E Group, and vegetable oil treatment on Model Group and Control Group for 20 days had no significant effects on body weights of different group rats, with the body weight of Model Group and SDTL-E Group still significantly higher than the Control Group (*p* < 0.01) (Table 2 and Figure 1).

**TABLE 2:** Body weight of rats before ovariectomy and after treatment.

Groups	(*n*)	Body weight before ovariectomy (g)	Body weight after treatment day 1 (g)	Body weight after treatment day 20 (g)
Model	12	266.00 ± 10.37	341.92 ± 34.36**	343.00 ± 34.93**
Control	12	268.58 ± 11.02	289.08 ± 21.04	291.25 ± 21.78
SDTL-E	10	264.80 ± 8.16	346.00 ± 29.62**	341.10 ± 28.51**

Values are mean ± SD.

**p < 0.01 vs. Control Group.

### SDTL-E treatment reduced serum alkaline phosphatase activity of ovariectomized-induced osteoporosis rats

**FIGURE 2:**
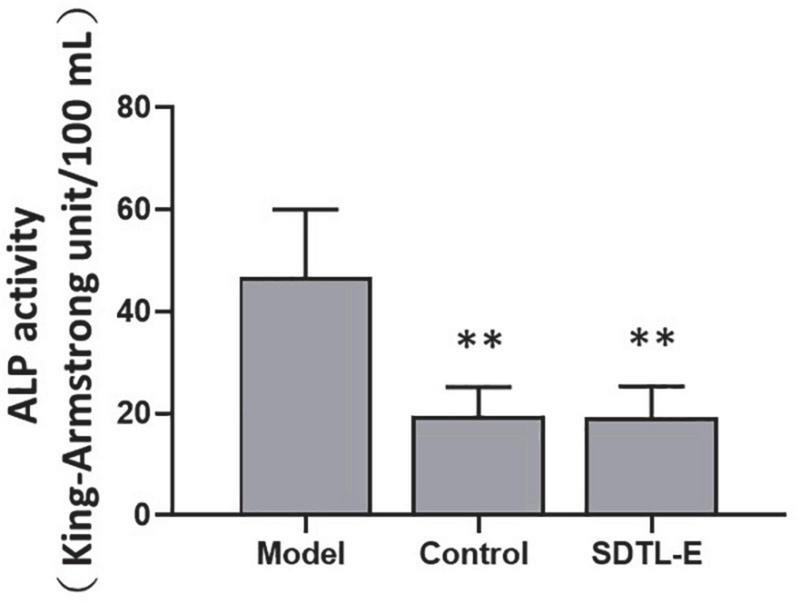
SDTL-E treatment reduced serum ALP activity of OVX-induced osteoporosis rats. Model Group (*n* = 11) and Control Group (*n* = 10) rats were treated with vegetable oil. SDTL-E Group (*n* = 10) rats were treated with SDTL-E. All treatments were topically applied twice daily for 20 days. Serum ALP activities were determined by ALP assay. Values are mean ± *SD*. ^**^p < 0.01 vs. Model Group.

ALP assay was performed to evaluate the effect of SDTL-E on the bone turnover biomarker, serum ALP activity. Compared to Control Group with serum ALP activity of 19.41 ± 5.76 King-Armstrong unit (K.A.U)/100 mL, Model Group had a 140.54% significantly higher serum ALP activity of 46.69 ± 13.32 K.A.U/100 mL (*p* < 0.01). After 20 days of SDTL-E treatment, serum ALP activity of SDTL-E Group rats was 19.24 ± 6.06 K.A.U/100 mL, significantly lower than that of the Model Group by 58.79% (*p* < 0.01) and was similar to that of the Control Group (Figure 2), indicating topical treatment of SDTL-E for 20 days can significantly regulate and reduce serum ALP activity in OVX-induced osteoporosis rats.

### SDTL-E treatment increased serum estradiol level of ovariectomized-induced osteoporosis rats

**FIGURE 3:**
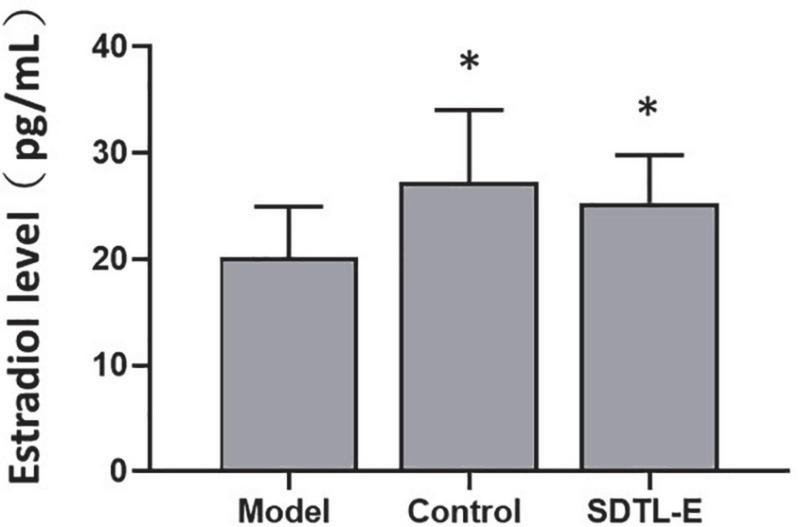
SDTL-E treatment increased serum estradiol level of OVX-induced osteoporosis rats. Model Group (*n* = 11) and Control Group (*n* = 11) rats were treated with vegetable oil. SDTL-E Group (*n* = 10) rats were treated with SDTL-E. All treatments were topically applied twice daily for 20 days. Serum estradiol levels were determined by estradiol assay. Values are mean ± *SD*. *p < 0.05 vs. Model Group.

Estradiol assay was performed to evaluate the effect of SDTL-E on serum estradiol level. Compared to Control Group with serum estradiol level of 27.27 ± 6.77 pg/mL, Model Group had a 26% significantly lower serum estradiol level of 20.18 ± 4.77 pg/mL (*p* < 0.05). After 20 days of SDTL-E treatment, serum estradiol level of SDTL-E Group rats was 25.30 ± 4.52 pg/mL, significantly higher than that of the Model Group by 25.37% (*p* < 0.05) and was similar to that of the Control Group (Figure 3), indicating topical treatment of SDTL-E for 20 days can significantly increase and restore serum estradiol level in OVX-induced osteoporosis rats.

### SDTL-E treatment reduced serum and urinary calcium ion levels of ovariectomized-induced osteoporosis rats

**FIGURE 4:**
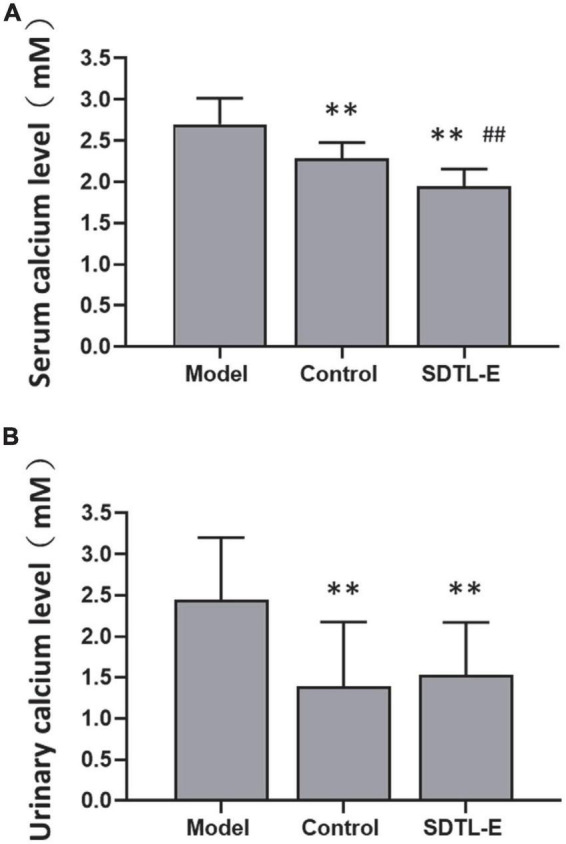
SDTL-E treatment reduced serum and urinary calcium ion levels of OVX-induced osteoporosis rats. Model Group (*n* = 11–12) and Control Group (*n* = 10–11) rats were treated with vegetable oil. SDTL-E Group (*n* = 10) rats were treated with SDTL-E. All treatments were topically applied twice daily for 20 days. Calcium ion levels in **(A)** serum and **(B)** urine were determined by calcium assay. Values are mean ± *SD*. ^**^p < 0.01 vs. Model Group. ^##^*p* < 0.01 vs. Control Group.

Calcium assay was performed to evaluate the effects of SDTL-E on the bone turnover biomarkers, serum and urinary calcium ion levels. Compared to Control Group with serum calcium ion level of 2.28 ± 0.20 mM, Model Group had a 18.42% significantly higher serum calcium ion level of 2.70 ± 0.32 mM (*p* < 0.01). After 20 days of SDTL-E treatment, serum calcium ion level of SDTL-E Group rats was 1.94 ± 0.21 mM, significantly lower than that of the Model Group by 28.15% (*p* < 0.01) and was also lower than that of the Control Group (*p* < 0.01) (Figure 4A), indicating topical treatment of SDTL-E for 20 days can significantly stop bone calcium loss and the transfer of calcium ions to the blood in OVX-induced osteoporosis rats.

Compared to Control Group with urinary calcium ion level of 1.39 ± 0.79 mM, Model Group had a 76.26% significantly higher urinary calcium ion level of 2.45 ± 0.76 mM (*p* < 0.01). After 20 days of SDTL-E treatment, urinary calcium ion level of SDTL-E Group rats was 1.76 ± 0.80 mM, significantly lower than that of the Model Group by 28.16% (*p* < 0.01) and was similar to that of the Control Group (Figure 4B), indicating topical treatment of SDTL-E for 20 days can significantly stop the loss and transfer of calcium ions to the urine in OVX-induced osteoporosis rats.

### SDTL-E treatment had no significant effects on urinary calcium/creatinine ratio of ovariectomized-induced osteoporosis rats

**FIGURE 5:**
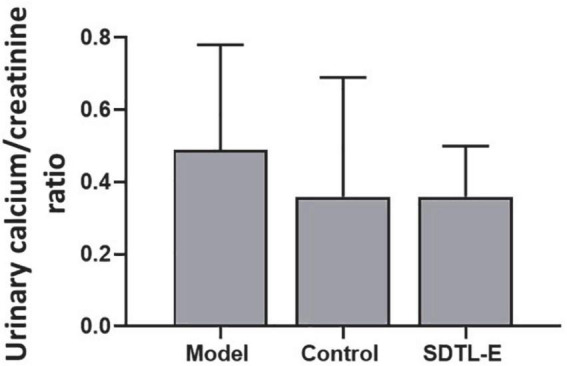
SDTL-E treatment had no significant effects on urinary calcium/creatinine ratio of OVX-induced osteoporosis rats. Model Group (*n* = 12) and Control Group (*n* = 11) rats were treated with vegetable oil. SDTL-E Group (*n* = 10) rats were treated with SDTL-E. All treatments were topically applied twice daily for 20 days. Urinary calcium/creatinine ratio was calculated by results obtained from calcium assay and creatinine assay. Values are mean ± *SD*.

Calcium assay and creatinine assay were performed to quantify urinary calcium and creatinine to calculate urinary calcium/creatinine ratios in different rat groups. Compared to Control Group with urinary calcium/creatinine ratio of 0.36 ± 0.33, Model Group had a 36.11% higher urinary calcium/creatinine ratio of 0.49 ± 0.29, but the difference was not statistically significant. After 20 days of SDTL-E treatment, urinary calcium/creatinine ratio of SDTL-E Group rats was 0.36 ± 0.14, which was similar to that of the Control Group and was lower than that of the Model Group, but the difference was not statistically significant (Figure 5).

### SDTL-E treatment had no significant effects on bone mineral density of ovariectomized-induced osteoporosis rats

**FIGURE 6:**
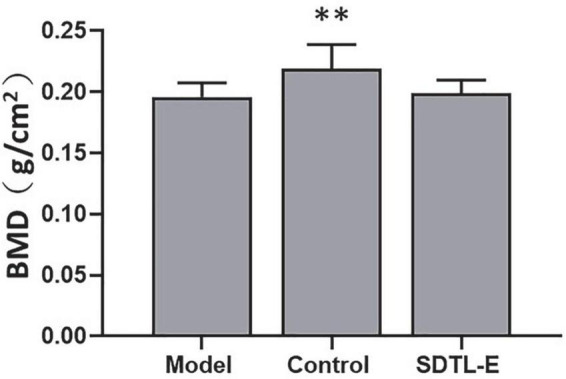
SDTL-E treatment had no significant effects on BMD of OVX-induced osteoporosis rats. Model Group (*n* = 12) and Control Group (*n* = 11) rats were treated with vegetable oil. SDTL-E Group (*n* = 10) rats were treated with SDTL-E. All treatments were topically applied twice daily for 20 days. BMD was determined by performing bone densitometry evaluation on the right femur. Values are mean ± *SD*. ^**^p < 0.01 vs. Model Group.

Bone densitometry was performed on the right femur to evaluate the effects of SDTL-E on BMD. Compared to Control Group with BMD of 0.22 ± 0.02 g/cm^2^, Model Group had a 9.09% significantly lower BMD of 0.20 ± 0.01 g/cm^2^ (*p* < 0.01). After 20 days of SDTL-E treatment, BMD of SDTL-E Group rats was 0.20 ± 0.01 g/cm^2^, which had no significant difference with the Model Group (Figure 6), indicating topical treatment of SDTL-E for 20 days has no significant effect on BMD in OVX-induced osteoporosis rats.

### SDTL-E treatment increased bone strength of ovariectomized-induced osteoporosis rats

**FIGURE 7:**
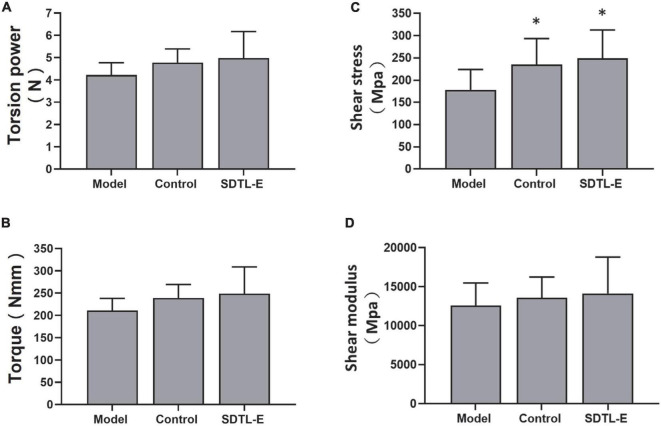
SDTL-E treatment increased bone strength of OVX-induced osteoporosis rats. Model Group (*n* = 9) and Control Group (*n* = 11) rats were treated with vegetable oil. SDTL-E Group (*n* = 9) rats were treated with SDTL-E. All treatments were topically applied twice daily for 20 days. Bone mechanics parameters, **(A)** torsion power **(B)** torque **(C)** shear stress and **(D)** shear modulus were determined by bone torsional testing. Values are mean ± *SD*. *p < 0.05 vs. Model Group.

Bone torsional testing results showed shear stress of Model Group was significantly lower than that of the Control Group (*p* < 0.05) (Supplementary Table 1 and Figure 7C). Torsion power, torque and shear modulus of Model Group were lower than that of the Control Group, but the differences were not statistically significant (Supplementary Table 1 and Figures 7A,B,D). Compared to Model Group with shear stress of 178.281 ± 45.672 Mpa, shear stress of SDTL-E Group rats after 20 days of SDTL-E treatment was 249.502 ± 63.445 Mpa, significantly higher than that of the Model Group by 39.93% (*p* < 0.05) and was similar to the shear stress of the Control Group of 235.540 ± 58.097 Mpa (Supplementary Table 1 and Figure 7C). Torsion power, torque and shear modulus of SDTL-E Group were similar to that of the Control Group and were higher than that of the Model Group but the differences were not statistically significant (Supplementary Table 1 and Figures 7A,B,D). These results indicate that topical treatment of SDTL-E for 20 days can increase bone strength in OVX-induced osteoporosis rats.

### SDTL-E treatment improved the structure of osseous tissue of ovariectomized-induced osteoporosis rats

**FIGURE 8:**
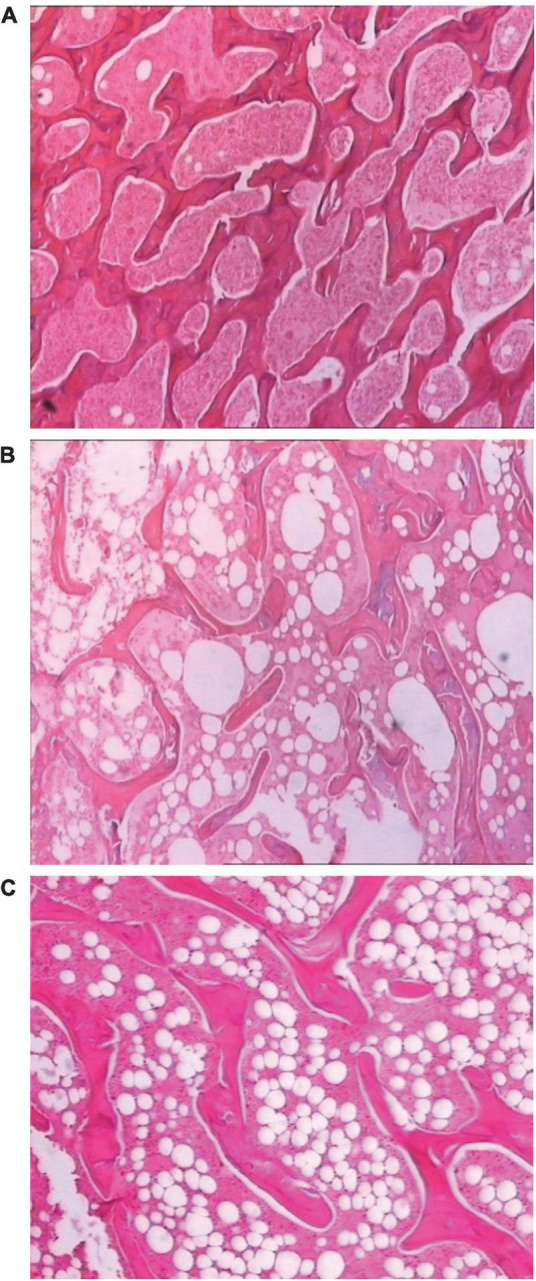
SDTL-E treatment improved the structure of osseous tissue of OVX-induced osteoporosis rats. Model Group and Control Group rats were treated with vegetable oil. SDTL-E Group rats were treated with SDTL-E. All treatments were topically applied twice daily for 20 days. Osseous tissues of left femur were stained by hematoxylin and eosin, followed by observation under light microscope with 200X magnification. Above are representative staining images of **(A)** Control Group **(B)** Model Group and **(C)** SDTL-E Group rats.

Figure 8A shows a clear structure of osseous tissue of the Control Group rats, the trabecular bones were thick, darkly stained and densely aligned, the interspaces of trabeculae were small and filled with red bone marrow indicating active blood production. Figure 8B shows the structure of osseous tissue of the Model Group rats, when compared with the Control Group rats, the number of trabecular bones decreased, the trabecular bones were lightly stained, shrunk, fractured and sparse, the interspaces of trabeculae were widened, fatty tissue increased with fewer red bone marrow indicating low blood production. Figure 8C shows the structure of osseous tissue of the SDTL-E Group rats, when compared with the Control Group rats, the number of trabecular bones decreased, the trabecular bones were lightly stained, shrunk, fractured and sparse, the interspaces of trabeculae were widened, fatty tissue increased with fewer red bone marrow indicating low blood production, however, when compared with the Model Group rats, the number of trabecular bones and trabecular bone thickness showed a slight increase, studies with longer duration will be needed to investigate longer term effects of SDTL-E on osseous tissue structure of OVX-induced osteoporosis rats.

## Discussion

In postmenopausal osteoporosis, estrogen deficiency leads to increased bone turnover, which can be indicated by an elevation of the bone turnover marker, serum ALP activity (23). Despite an overall increase in bone turnover, bone resorption exceeds bone formation as indicated in a study that menopause induced 37–52% and 79–97% increase in bone formation and bone resorption marker levels, respectively (24), resulting in increased net bone resorption, which is the main determinant of high serum calcium levels (25). Lowered estrogen levels also lead to increased calcium excretion through kidney and decreased intestinal absorption of calcium (26), which stimulates bone calcium release into blood (25). These factors above lead to observations that the bone turnover markers serum calcium, urinary calcium and urinary calcium/creatinine are elevated in postmenopausal osteoporosis patients (27, 28). Results of this study showed topical treatment of SDTL-E for 20 days on SDTL-E Group rats resulted in significantly higher estradiol, lower serum ALP, lower serum calcium and lower urinary calcium levels when compared to Model Group rats. These results suggest that SDTL-E can regulate and restore estradiol levels to reduce bone turnover, net bone resorption, bone calcium loss and excretion of calcium through kidney in OVX-induced osteoporosis rats.

Apart from being a bone turnover marker, serum ALP is also a marker for bone formation by osteoblasts (24). SDTL-E lowering serum ALP indicated suppression of bone turnover and bone formation by osteoblasts, and SDTL-E lowering serum calcium level indicated a decrease in net bone resorption, these observations indirectly reflect that SDTL-E also suppressed bone resorption by osteoclasts and this suppression effect is greater than that on bone formation by osteoblasts in OVX-induced osteoporosis rats. However, the results did not individually evaluate how bone formation and bone resorption were affected by SDTL-E. Future studies with evaluation of changes on bone formation markers, such as serum osteocalcin, and bone resorption markers, such as serum carboxyterminal cross-linked telopeptide of type I collagen (CTX), and analysis of osteoclast activation by tartrate-resistant acid phosphatase (TRAP) staining can be done to give a full picture on how SDTL-E reduces net bone resorption.

Estradiol is the most common and biologically active form of estrogen in mammals. Apart from the ovaries, estradiol is also produced in various extragonadal organs, such as the pancreas, brain, adrenal glands, skin and adipose tissue (29, 30). It is important to note that high levels of estrogen not only increase the risk of developing breast and endometrial cancer (15), but can also lead to side effects such as thrombosis (31). The results of this study suggest, SDTL-E, which consists of only plant extracts, probably stimulated estradiol production from the above-mentioned organs to endogenously restore estradiol levels in OVX-induced osteoporosis rats back to normal levels similar to that of Control Group rats, therefore minimizing the risks of causing side effects related to high estrogen levels.

Bone strength is positively correlated to bone’s ability to resist fracture (32). The commonly used marker of bone strength, BMD (33), as well as bone mechanics indicators from torsional testing, were assessed in this study to investigate SDTL-E effect on bone strength of OVX-rats. BMD of Model Group OVX-rats was significantly lower than that of Control Group rats, indicating successful establishment of the OVX-induced osteoporosis rat model. Bone torsional experiments showed SDTL-E treatment on SDTL-E Group rats resulted in significantly higher shear stress compared to Model Group, torsion power, torque and shear modulus of SDTL-E Group rats were also increased and restored to similar levels to that of Control Group rats, indicating that topical treatment of SDTL-E can restore bone strength of OVX-rats back to near normal level in 20 days. On the other hand, treatment of SDTL-E for 20 days on SDTL-E Group rats did not have significant effects on BMD. This could be due to the duration of the study is much shorter than the time needed for mineralization of newly formed bone, which can take up to around 30 months (32). However, that does not indicate SDTL-E cannot improve bone strength as BMD only accounts for 50–70% of the variation in bone strength (32, 34).

BMD and bone structure both contribute to the strength of the skeleton (32). The early stage of the bone remodeling process starts from the trabecular bone. The number of trabecular bones, trabecular bone thickness and the degree of connectivity, which are decreased in osteoporosis, influence the mechanical strength of the bone (33). SDTL-E treatment on OVX-rats for 20 days restored bone strength but not BMD back to normal levels, suggesting that SDTL-E increased bone strength of OVX-rats through mechanisms other than restoring BMD. Microscopic observations from this study demonstrated Model Group and SDTL-E Group rats had decreased, shrunk, fractured and sparse trabecular bone structure compared to Control Group rats, however, number of trabecular bones in SDTL-E Group rats was greater than that in Model Group rats, indicating that topical treatment of SDTL-E for 20 days can already increase bone strength of OVX-rats through improving trabecular bone number and thickness. Despite the calcium content is low in trabecular bone, the increase in number of trabecular bones in SDTL-E Group rats can also be accounted for the reduction of serum calcium level by SDTL-E as calcium is required for trabecular bone formation (35). While current bone histology results provided qualitative analysis on observable changes of trabecular bone structure, future experiments with more in-depth analysis using imaging software to measure bone histomorphometric data of stained trabecular tissues or employing micro computed X-ray tomography (microCT) to evaluate the 3D bone microstructure can be done to accurately quantify the improvements of trabecular bone structure by SDTL-E treatment.

Osteoporotic fractures can lead to increased mortality rate (5), which might have been caused by post-fracture complications. The major complications related to post-fracture deaths include cardiac, respiratory, cerebrovascular, and malignancy diseases (16). The study results showed topical treatment of SDTL-E increased and restored bone strength in 20 days, indicating SDTL-E can prevent fractures and therefore reduce post-fracture mortality rate. Histological observations showed Model Group OVX-rats had significantly increased body weight, decreased trabecular bone number, increased fatty tissue and decreased blood producing red bone marrow between interspaces of trabeculae when compared to Control Group rats. Previous studies showed estrogen deficiency in post-menopause can lead to obesity and increased fat in bone marrow (36), positive correlation between blood cell count and BMD in post-menopausal women (37), and anemia is associated with increased risk of osteoporosis and fracture risk (38, 39). Combined observations from the above studies and the current study suggest that estrogen deficiency leading to obesity and increased fatty tissue between interspaces of trabeculae resulting in anemia is a possible mechanism for developing osteoporosis in OVX-rats. SDTL-E treatment for 20 days on SDTL-Group rats restored estradiol level, bone strength and trabecular bone structure, but did not decrease body weight or increase blood producing red bone marrow. Contrary observations were found in previous studies demonstrating that estrogen can regulate body weight (40) and increase red blood cell production (41). It was a drawback of this study to have not quantitatively monitored the amount of blood producing red bone marrow and therefore could not assess the effect of restored estradiol level on the changes of blood producing red bone marrow, which can be investigated in future experiments with longer study duration and quantification of red bone marrow by dual-energy computed tomography (42), or through measuring the amount of hematopoietic cellular constituents in bone marrow by flow cytometry (43).

Some studies have shown that other osteoporosis drugs administered through oral gavage or injection in OVX-induced osteoporosis rats require longer time to restore bone strength than topical application of SDTL-E, and their efficacy may not be significant. For example, oral gavage of the bisphosphonate drug, Alendronate Sodium, took 6 months (∼180 days) to significantly increase and restore bone strength back to normal level (44). Injection of the parathyroid hormone analog drug, Forteo^®^, took 12 weeks (∼84 days) to significantly increase and restore bone strength back to normal level (45). Oral gavage of the selective estrogen receptor modulator drug, Raloxifene for 4 weeks (∼28 days) did not significantly increase bone strength (46). For other herbal preparation or plant extracts, studies on their topical application effects on bone strength in OVX-rats are lacking, but reports have shown they can restore bone strength in OVX-induced osteoporosis rats after oral administration for a period ranging from 4 weeks (∼28 days) (47) to 26 weeks (∼182 days) (48). This study demonstrated that SDTL-E can treat osteoporosis in OVX-rats by increasing bone strength, number of trabecular bones and trabecular bone thickness. Its possible mechanism is through stimulation of endogenous regulation and restoration of estradiol back to normal level to reduce bone turnover, net bone resorption, bone calcium loss and excretion of calcium through kidney. The above-mentioned therapeutic effects can be achieved by topical application of SDTL-E for 20 days, which is shorter than the time required by the above-mentioned osteoporosis drugs, herbal preparation or plant extracts to restore bone strength (44–48), and much shorter than the 6 months-time for post-fracture deaths to occur reported in other studies (16). Therefore, SDTL-E can effectively treat osteoporosis and restore bone strength for preventing fractures, lowering fracture rate and post-fracture mortality rate, improving the clinical efficacy for osteoporosis.

While this study demonstrated the effects of SDTL-E in treating OVX-induced osteoporosis rats, improvements can be done to the study design by adding a Control Group with SDTL-E treatment, a Positive Control Group with a known osteoporosis drug treatment and SDTL-E Treatment Groups with different SDTL-E dosages. These improvements can be implemented in future studies to provide valuable information on SDTL-E effects on bones of normal rats or participants, how SDTL-E performs when directly compared to other anti-osteoporotic drugs and the optimal dosage of SDTL-E to be used in treating osteoporosis.

The current study findings suggest topical application of SDTL-E has the advantages of fast and obvious therapeutic effects, and has potential to be developed into osteoporosis drug with quick efficacy and minimal side effects. These findings may overthrow the common perception that the onset time of topical drugs are slower than that of oral and injection drugs, providing new insights for future drug research.

## Conclusion

Topical treatment of SDTL-E for 20 days improved bone strength and trabecular bone structure in OVX-induced osteoporotic rats. The underlying mechanisms include restoring estradiol back to normal level, reducing bone turnover, net bone resorption, bone calcium loss and excretion of calcium through kidney. These results suggest SDTL-E has the potential to be developed into osteoporosis drug with quick efficacy and minimal side effects, and topical application of natural plant extract is a possible new approach for treatment of osteoporosis.

## Data availability statement

The original contributions presented in this study are included in the article/Supplementary material, further inquiries can be directed to the corresponding author/s.

## Ethics statement

The animal study was reviewed and approved by the Laboratory Animal Ethics Committee of Jinan University and was performed in accordance with Animal Care Ethical Guidelines of Jinan University.

## Author contributions

H-YS, J-HL, and A-HX: conceptualization and methodology. H-YS, J-HL, A-HX, and BW: data analysis. JT: funding acquisition. J-HL and A-HX: investigation. H-YS: supervision. BW: writing—original draft. H-YS, JT, and BW: writing—review and editing. All authors have read and agreed to the published version of the manuscript.
