# Supplementary material for: Topical application of the plant extract SDTL-E in ovariectomized rats: A potential new approach for treating osteoporosis
Source: Front Med (Lausanne). 2022 Oct 20;9:988235. doi: 10.3389/fmed.2022.988235 (PMC9631791; doi:10.3389/fmed.2022.988235)
Supplement: Supplementary file 1 [file Table_1.DOCX]

**Table S1.** Bone torsional testing results. Model Group and Control Group rats were treated with vegetable oil. SDTL-E Group rats were treated with SDTL-E. All treatments were topically applied twice daily for 20 days. Values are mean ± SD. **p*<0.05 vs Model Group.

| Groups | (n) | Bone mechanics indicators | | | | |
| --- | --- | --- | --- | --- | --- | --- |
|  |  | Torsion power  (N) | Torque  (Nmm) | Shear stress  (MPa) | Shear modulus  (MPa) |  |
| Model | 9 | 4.22±0.55 | 210.94±27.31 | 178.281±45.672 | 12580.98±2903.18 |  |
| Control | 11 | 4.78±0.61 | 238.94±30.51 | 235.540±58.097* | 13604.84±2646.92 |  |
| SDTL-E | 9 | 4.98±1.19 | 249.03±59.75 | 249.502±63.445* | 14160.41±4643.71 |  |
